# Genomic insights and advanced machine learning: characterizing autism spectrum disorder biomarkers and genetic interactions

**DOI:** 10.1007/s11011-023-01322-3

**Published:** 2023-12-28

**Authors:** Laila Dabab Nahas, Ankur Datta, Alsamman M. Alsamman, Monica H. Adly, Nader Al-Dewik, Karthik Sekaran, K Sasikumar, Kanika Verma, George Priya C Doss, Hatem Zayed

**Affiliations:** 1 Biosciences Department, Durham University, Durham, UK; 2 Laboratory of Integrative Genomics, Department of Integrative Biology, School of Bio Sciences and Technology, Vellore Institute of Technology (VIT), Vellore, Tamil Nadu 632014 India; 3 Agricultural Genetic Engineering Research Institute (AGERI), Agricultural Research Center (ARC), Giza, Egypt; 4 Department of Research, Women’s Wellness and Research Center, Hamad Medical Corporation, Doha, Qatar; 5 Center for Brain Research, Indian Institute of Science, Bengaluru, India; 6 Department of Sensor and Biomedical Technology, School of Electronics Engineering, Vellore Institute of Technology, Vellore, Tamil Nadu India; 7 Department of parasitology and host biology ICMR-NIMR, Dwarka, Delhi India; 8 Department of Biomedical Sciences College of Health Sciences, QU Health, Qatar University, Doha, Qatar

**Keywords:** Autism spectrum disorder, Single nucleotide polymorphism, Artificial Intelligence, Pathway Enrichment Analysis, Multi-omics, SHapley Additive exPlanations

## Abstract

Autism Spectrum Disorder (ASD) is a complex neurodevelopmental condition characterized by altered brain connectivity and function. In this study, we employed advanced bioinformatics and explainable AI to analyze gene expression associated with ASD, using data from five GEO datasets. Among 351 neurotypical controls and 358 individuals with autism, we identified 3,339 Differentially Expressed Genes (DEGs) with an adjusted p-value (≤ 0.05). A subsequent meta-analysis pinpointed 342 DEGs (adjusted p-value ≤ 0.001), including 19 upregulated and 10 down-regulated genes across all datasets. Shared genes, pathogenic single nucleotide polymorphisms (SNPs), chromosomal positions, and their impact on biological pathways were examined. We identified potential biomarkers (*HOXB3, NR2F2, MAPK8IP3, PIGT, SEMA4D*, and *SSH1*) through text mining, meriting further investigation. Additionally, ‎we shed light on the roles of *RPS4Y1* and *KDM5D* genes in neurogenesis and neurodevelopment. Our analysis detected 1,286 SNPs linked to ASD-related conditions, of which 14 high-risk SNPs were located on chromosomes 10 and X. We highlighted potential missense SNPs associated with *FGFR* inhibitors, suggesting that it may serve as a promising biomarker for responsiveness to targeted therapies. Our explainable AI model identified the *MID2* gene as a potential ASD biomarker. This research unveils vital genes and potential biomarkers, providing a foundation for novel gene discovery in complex diseases.

## Introduction

Autism Spectrum disorder (ASD) is a neurodevelopmental disorder mainly affecting the brain, immune system, and gastrointestinal tract (Chow et al. 2012). Its characteristics include and are not limited to restricted interests, repetitive behaviors, and social communication disorders (Alonso-Gonzalez et al. 2018). ASD is generally considered a multifactorial disorder with genetic effects and non-genetic components of risk. The exact cause of ASD has not been fully defined, but a strong genetic component has been demonstrated through familial studies (Eissa et al. 2018). In addition, genetic studies have found that alterations within the developmental pathways of the neuronal and axonal systems appear to be strongly involved in synaptogenesis due to single-gene mutations (Eissa et al. 2018). Microarray is an important first-line technique to reveal the genetic contribution to ASD and other complicated neurobehavioral disorders (Mehta et al. 2010; Sarachana and Hu 2013; Benítez-Burraco 2020). This method has been used to study the pathology of ASD and to detect differentially expressed genes (DEGs) among individuals who are autistic and normal (Kuwano et al. 2011; Sekaran et al. 2021; Voineagu et al. 2011; Hu and Lai 2013; Sarachana and Hu 2013). Although microarray technology is a strategy for identifying associated genes and underlying biological mechanisms, genes defined in one study may not be detected in others (Zhang et al. 2017). The reliability and generalization of results can be improved by combining information from multiple reported studies and datasets (Ramasamy et al. 2008; Udhaya Kumar et al. 2021; Datta et al. 2023). The study of complicated disorders such as ASD requires a background understanding of their pathogenesis, evolutionary history, and mapping of genetic loci using an integrated analysis. Network analysis for autism-related genes through protein-protein interactions (PPIs) is an alternative method to evaluate the dynamic influences of associated candidate genes. Such an assessment can suggest a list of gene-drug targets (Corominas et al. 2014).

Recently, next-generation sequencing (NGS) techniques have transformed the capacity of researchers and clinicians to gather genetic data. Moreover, machine learning (ML) methods have been integrated with NGS in recent years to revolutionize bioinformatics tools and approaches. (Hassan et al. 2022). Numerous literature assesses and showcases the various applications of ML and AI in disease and drug research (Li et al. 2021). Databases dedicated to understanding the molecular genetics of diseases serve as valuable tools for investigating the epidemiology of ASD, providing comprehensive insights into the clinical manifestations and genetic backgrounds of individuals with ASD (Tye et al. 2018). To understand the genetic etiology of ASD, it is beneficial to employ an integrated, multidisciplinary approach. Modern bioinformatics techniques are instructive for deciphering ASD data. Additionally, computational research has shed light on the underlying mechanisms of ASD, confirming the importance of such tools in understanding this complex disorder (Rosenberg et al. 2015; Ray et al. 2019). Furthermore, numerous computational studies on various pathogenesis have been reported (Habib et al. 2020; Younes et al. 2020; Micheal et al. 2020). Utilizing diverse datasets and advanced bioinformatics tools, we have achieved a holistic understanding of ASD. We examined genetic variants across the different GEO datasets, patients with ASD, and normal controls and identified pathogenic SNPs linked to ASD genes. Our exploration extended to recognizing consistently associated SNPs or genes, understanding protein-protein interactions, and analyzing gene pathways and ontologies related to ASD. Additionally, we harnessed text-mining tools to gauge gene frequency in ASD literature and employed the SHAP model to uncover potential ASD biomarkers.

## Materials and methods

### Dataset information

The dataset used for this analysis can be accessed through the Gene Expression Omnibus database (GEO) with IDs GSE29918, GSE29691, GSE37772, GSE111175, and GSE42133 (Luo et al. 2012; Pramparo et al. 2015a, b; Gazestani et al. 2019). These data were based on multiple platforms and the same cell types, showing gene expression profiles of 709 samples (Lymphoblastoid cell lines or leukocytes) isolated from 351 normal controls and 358 autistic individuals. The detection and quantification of DEGs in the transcription profiles were evaluated using the ImaGEO tool with standard parameters and an adjusted p-value of 0.05, and the method used was Fisher (Toro-Domínguez et al. 2019).

### Protein-protein interactions (PPIs), text mining, and gene ontology analysis

A PPI network assessment was performed using the STRING database (Szklarczyk et al. 2017, 2019, 2021). This analysis will show the protein interaction between the studied ones. For the enrichment analysis, there are many tools to characterize the genes’ functions. Thus, all DEGs studied were entered into DAVID, Shinygo, and GOnet tools using the Entrez Gene ID to obtain enrichment categories, GO enrichment, and the pathways (Pomaznoy et al. 2018; Ge et al. 2020; Sherman et al. 2022). The ClinVar database was used to search for known disease-associated SNPs and their risk factors, wherein the common DEGs between the five datasets served as the input (Landrum et al. 2016). To detect the positions of the pathogenic SNPs in the genes, all the DEGs studied were entered into g: Profiler, a web server for functional enrichment analysis (Raudvere et al. 2019). Following this, only SNPs in delay or autism and intellectual or neurological diseases were submitted to SNPnexus and g: Profiler to annotate SNPs and genes according to effect and biological pathways (Dayem Ullah et al. 2018).

**Fig. 1:**
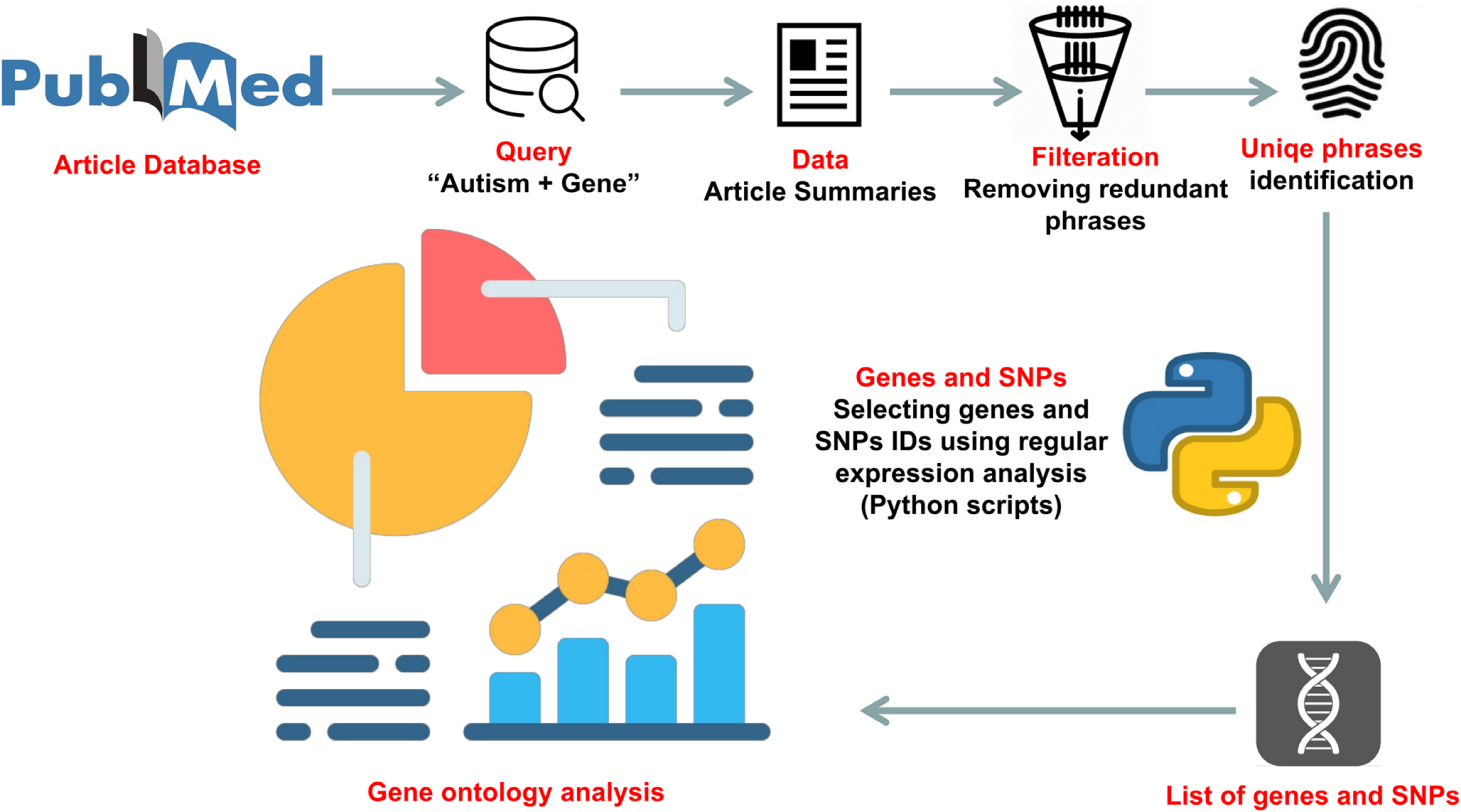
The text mining analysis pipeline used for studying some published autism literature

We performed text mining to identify previously reported genes associated with ‎ ASD. Our automated extraction process sourced data from many published ‎research related to ASD. From PubMed, 8,923 article summaries were downloaded that contained the query “autism + gene” from 2000 until May 1, 2021. In-home Python scripts were used to extract genes mentioned within the text. The complete text mining approach used for this study is shown in Fig. 1. Also, to find common genes between autism, schizophrenia, and other neural and brain disorders, we used DAVID PubMed results. For that, PubMed IDs resulting from DAVID were used to download the summaries of the articles until May 1, 2021. Then, in-house Python-based scripts were used to detect articles with autism, schizophrenia, and neural or brain, then extract genes mentioned within the text from each type of neurological disorder. The difference between the two text mining methods is that DAVID’s method searched the specific gene results in all published articles, not just in autism articles, like the first method.

### ASD gene biomarker identification by explainable artificial intelligence

Explainable Artificial Intelligence (XAI) offers significant potential for interpreting intricate representations amid diverse information sources. While ML algorithms are extensively employed to analyze biological data and identify potential disease biomarkers, the inherent black-box nature of traditional ML models complicates the understanding and interpretation of their decision-making processes (Lundberg and Lee 2017). XAI algorithms address these challenges by providing better insights into the evident decisions of the predictions by the AI models, ensuring accuracy, fairness, and transparency. These explanations assist researchers and experts in understanding the base factors and technical features the models consider when identifying biomarkers (Sekaran et al. 2023). Among the five datasets used in this study, we selected GSE42133 to perform biomarker experimentation based on a few selection criteria. The sample size, the number of classes in each dataset, and the differential expression analysis results were considered for selecting the dataset. The 56 control samples were assigned a binary value of 0, and the 91 affected patients’ samples were assigned a binary value of 1.

### Shapley additive explanations

SHapley Additive exPlanations (SHAP) is an XAI framework that interprets the predictions of machine learning models. It provides the contribution of every variable to the final prediction made by the model based on its importance. The method follows the cooperative game theory and the concept of Shapley values introduced by Lloyd Shapley in 1953 (Shapley 1953). SHAP describes the contribution of prediction by assigning a SHAP value to each feature of an input instance. SHAP value represents the fluctuation in the expected prediction in both cases where the particular feature is included compared to its exclusion impact (Lundberg et al. 2020). The following terms represent each parameter of the SHAP function, where X is the input features of a sample, f denotes the machine-learning model trained to predict the output, and φ is the SHAP value function.

The SHAP value function φ takes the following form:

$$\phi(X) = \phi_{0} + \phi_{1}(x_{1}) + \phi_{2}(x_{2}) + ... + \phi_{n}(x_{n})$$

In the above equation, φ_0_ represents the expected model output for a baseline reference. φ_i_(x_i_) represents the contribution of feature x_i_ to the model output. It identifies the change in the prediction when feature x_i_ is included compared to when it is excluded, considering all possible feature subsets.

## Results

### Identifying the differentially expressed genes (DEGs)

**Fig. 2:**
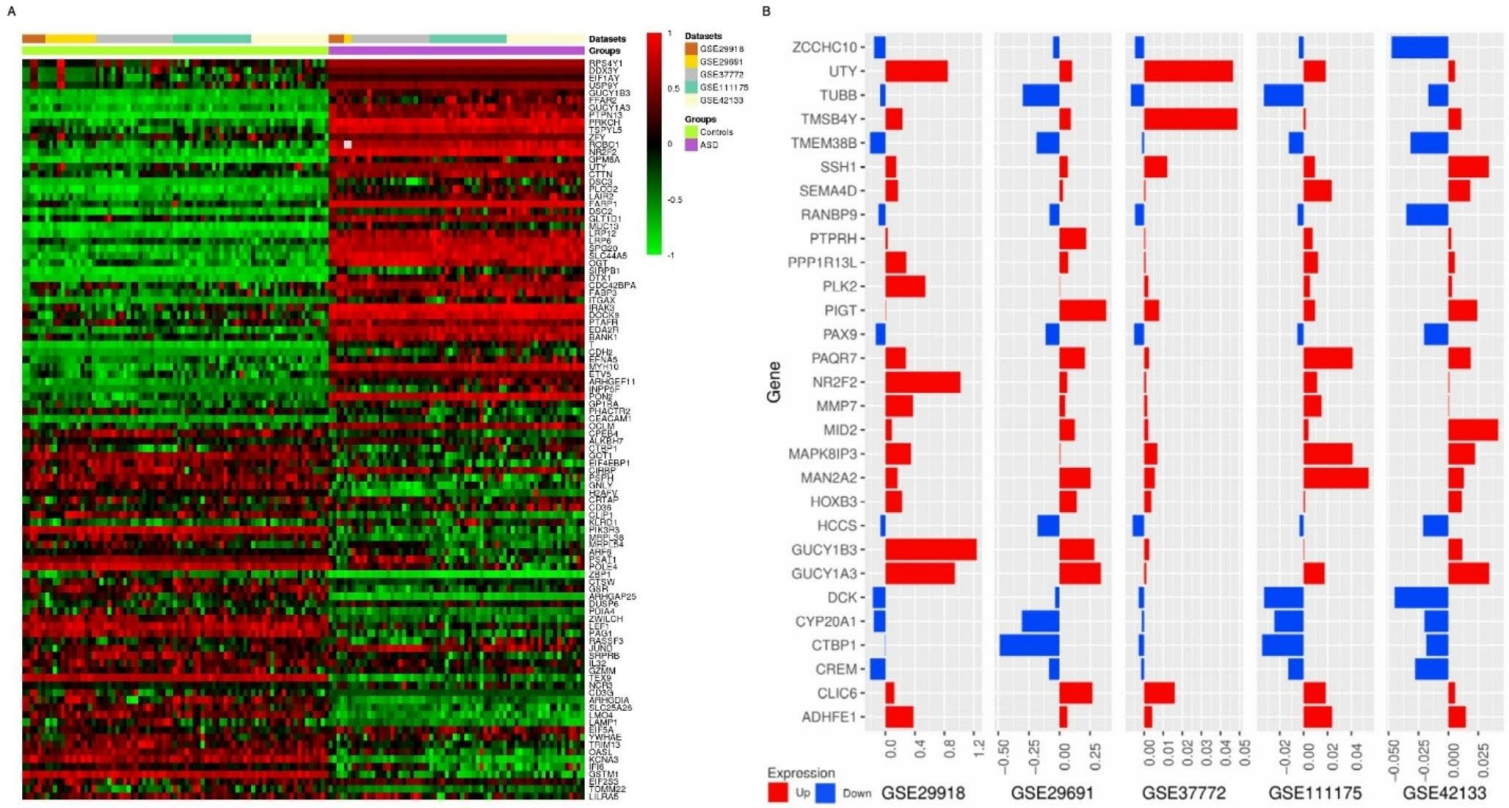
Aberrant expression of genes. (**A**) Complete heatmap of all DEGs. Two groups, ASD with purple and control with green, are discriminated clearly with down-regulated genes with green color and upregulated genes with red. (**B**) All common genes (up and down-regulated) between the five datasets. Up- and down-regulated genes with red and blue, respectively

Utilizing analytical samples from 351 neurotypical controls and 358 autism-affected subjects, 3,339 DEGs were identified with an adjusted p-value threshold of 0.05 of the five databases, all sourced from the same sample archetype, notably lymphoblastoid cell lines (LCL) and leukocytes. A heatmap of all DEGs was created (Fig. 2A). As shown in Fig. 2A, the heat map represents all DEGs over-expressed or under-expressed samples from each dataset. Meta-analysis results showed that 342 DGEs were found in all datasets with an adjusted p-value of 0.001, and these genes were used for the rest of the following analyses. Of them, 19 genes were upregulated, while 10 were down-regulated in all five datasets (Fig. 2B).

### PPI network analysis

**Fig. 3:**
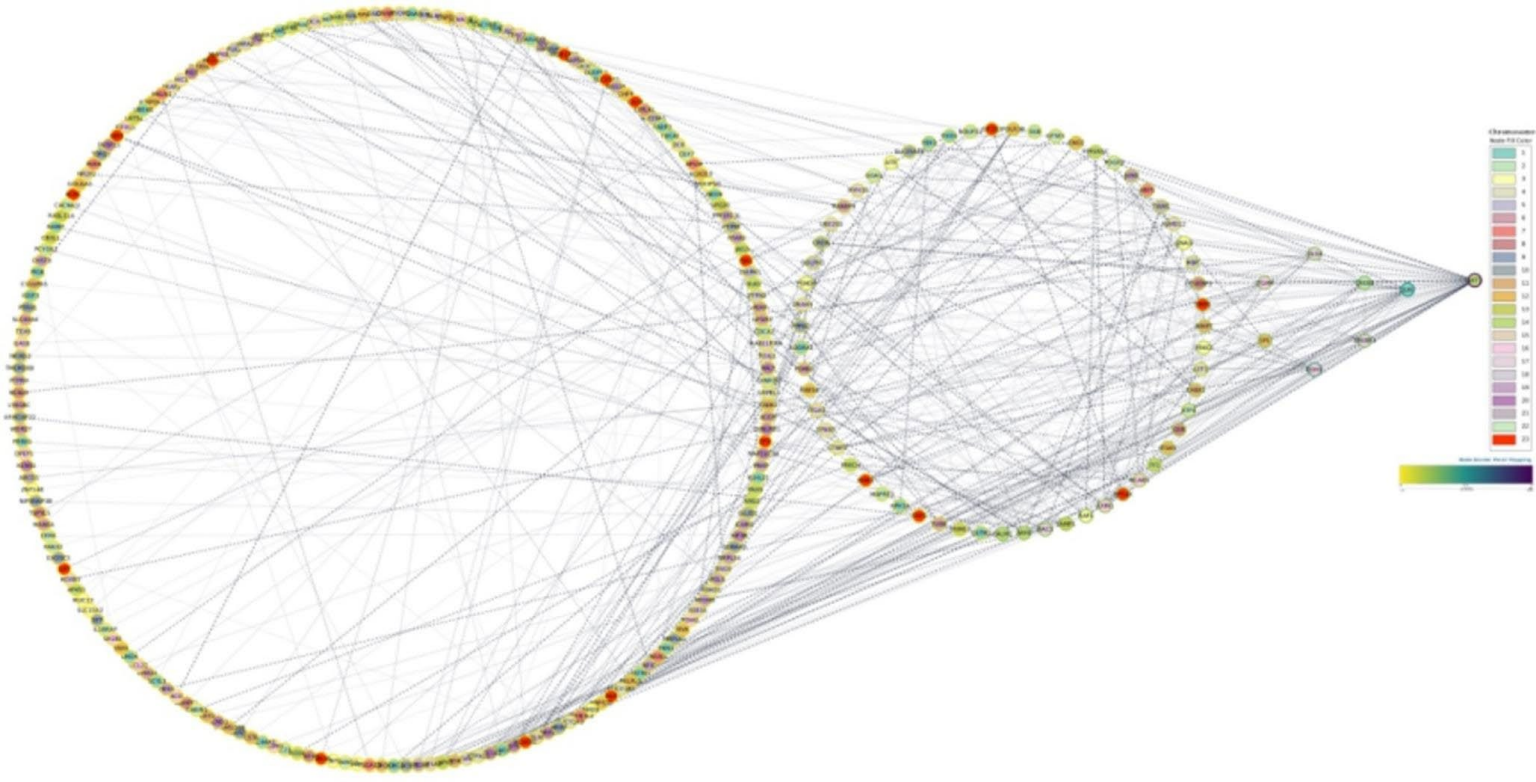
The interaction network of PPI for the top high-score genes

PPI networks are a graphical representation of the interactions between proteins within a biological system. The PPI shown in Fig. 3 exhibits the relationship found among most of the genes (261 out of 342) in this study. Moreover, about 44% of the genes in the network (115 genes) have more than 0.9 node scores. For example, *ATR, CHEK1, GUCY1A3, GUCY1B3*, and *MRPL34* are some of the genes with the highest node score, 0.999.

### Enrichment analysis and SNP analysis

**Fig. 4:**
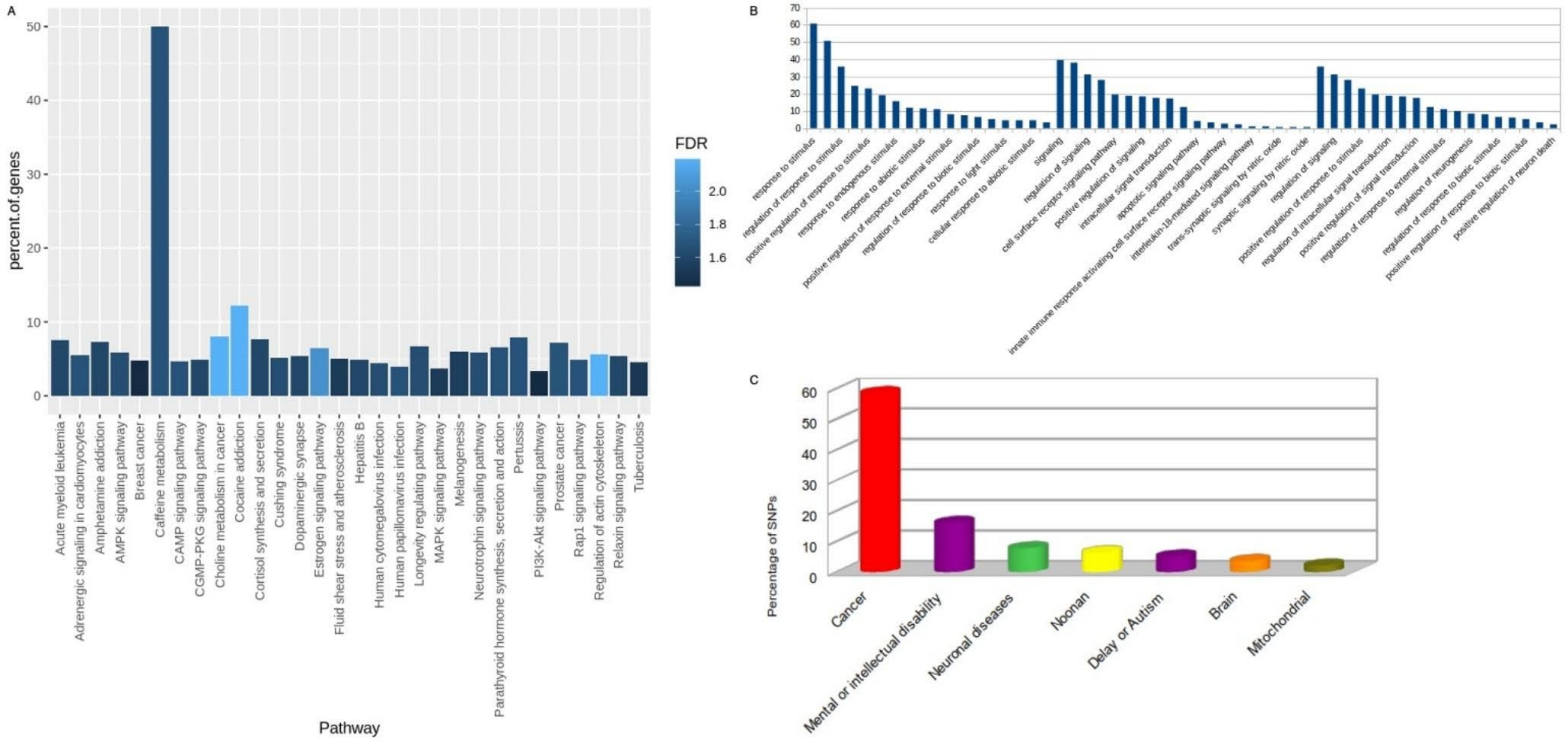
Functional Annotation of the DEGs. (**A**) Pathways in KEGG. (**B**) The biological process enrichment analysis. (**C**) All diseases related to the studied pathogenic SNPs.

**Fig. 5:**
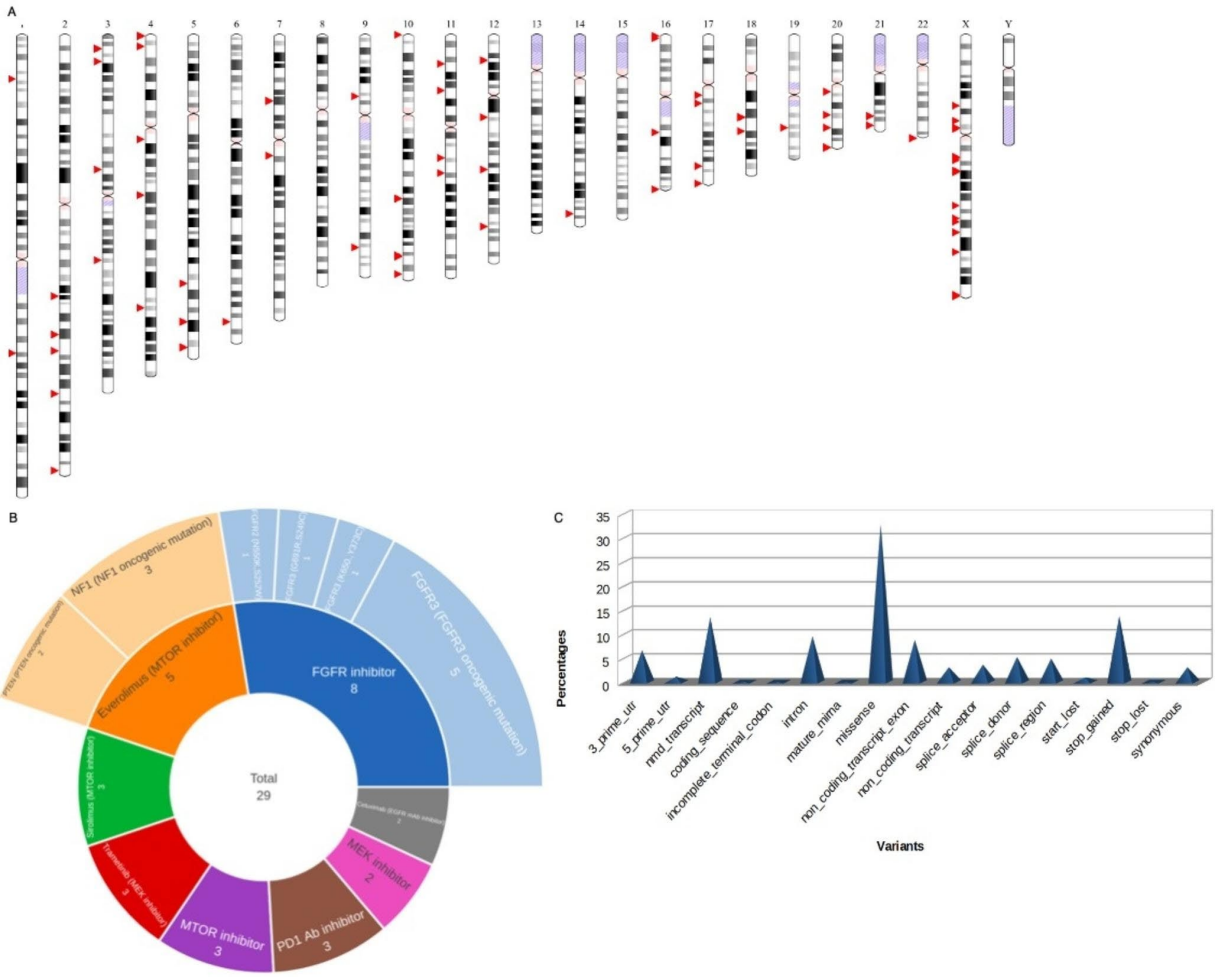
SNP analysis. (**A**) The positions of SNPs in autism and the most related diseases. (**B**) Biomarker sunburst chart for autism and the most related diseases SNPs. (**C**) The variant effects of autism and the most related diseases SNPs.

DAVID Functional Annotation, ShinyGo, and GOnet tools performed a functional enrichment analysis involving cytobands, chromosomes, diseases, and pathways for DEGs shared between the five datasets with adjusted p-value < 0.001. However, Gene Ontology (GO) enrichment was conducted for only genes involved in PPI’s interaction network. The results showed that chromosomes 19 and 17 have the highest number of genes, 31 and 30, respectively (Table 1). For cytobands, Xq13.1 and 22q13.1 have the highest numbers of genes (4 for each). Cancer (24%), pharmacogenomic (19.9%), and neurological (19.6%) were the most diseases found. Polymorphism (62.9%), alternative splicing (60.8%), and phosphoprotein (51.2%) were the highest keywords in our genes (Table 1). Also, according to DAVID-PubMed mining text results, we found 14, 16, and 211 genes related to autism, Schizophrenia, and neural or brain disorders. For the pathways, caffeine metabolism, cocaine addiction, and choline metabolism in cancer were found from the top pathways related to ASD genes, resulting in our study (Fig. 4A). The GO analysis from the biological process revealed that GO terms related to the responsibility to stimulus, signaling, and development or regulation of the nervous system have 171 (65%), 138 (53%), and 53 (20%) genes, respectively (Fig. 4B). ClinVar database was used to search for pathogenic SNPs, associated diseases, and associated risk factors (Fig. 4C). All pathogenic SNPs found, 753, 208, 100, 83, and 67, were associated with cancer, mental or intellectual, neuronal diseases, Noonan syndrome, and delay or autism, respectively (Fig. 4C). The positions of SNPs in autism and the most related diseases (delay, intellectual, and mental diseases) were detected on the GRCh38 Chromosomes (Fig. 5A). In addition, many non-synonymous variants were detected as potential biomarkers of response to targeted therapies for ASD, such as FGFR inhibitors (Fig. 5B). Also, the variant effects of the pathogenic SNPs were detected (Fig. 5C). These terms convey information about the effects each allele of the variant may have on each gene (Agrahari et al. 2018, 2019).‎.

**Table 1:** The five DEGs datasets and their details

Dataset name	Sample type	Platform	Total samples	Autism	Control	References
GSE29918	LCLs*	GPL5175	10	4	6	
GSE29691	LCLs	GPL570	15	2	13	
GSE37772	LCLs	GPL6883	439	233	206	(Luo et al. 2012)
GSE111175	leukocyte	GPL10558	98	28	70	(Gazestani et al. 2019)
GSE42133	leukocyte	GPL10558	147	91	56	(Pramparo, Lombardo, et al. 2015; Pramparo, Pierce, et al. 2015)

### Text mining

The text mining analysis revealed 3270 genes previously documented in the scientific literature discussing autism disorder-related genes. Among these genes were 50 genes common between our meta-analysis and the text mining from other articles. Moreover, 26 genes of the common 50 genes, such as *DLG4* (discs large MAGUK scaffold protein 4), *ATR* (ATR serine/threonine kinase), and *SH2B1* (SH2B adaptor protein 1), were found here in 0.9 of the PPI interaction networks. Furthermore, we tried to focus on the genes related to the development or regulation of the nervous system (53 genes), and using the results of text mining with articles published previously and PPI’s score > = 0.9, we could detect 13 genes that seem to be more involved in ASD’s disorders. These genes are *DLG4, MIF, ATR, TAF1, MED12, MBP, ATF4, ITGA3, CREB1, DSC2, EFNB3, YY1*, and *GDI1*.

### Dataset selection criteria

The bioinformatics analysis is conducted on GSE29918, GSE29691, GSE37772, GSE111175, and GSE42133 to identify molecular insights about ASD. An attempt has been made to develop machine-learning models for finding the gene biomarkers from these datasets. Initially, the datasets GSE29918 and GSE29691 are ruled out for their sample size of 14 and 15, respectively. In the next step, the total number of genes from each dataset is reduced during differential expression analysis. The datasets GSE37772 and GSE111175 listed only two (*RPS4Y1*, *KDM5D*) and zero genes, respectively, based on the criteria (adj. p-value < 0.01 & logFC > 0.5 or logFC < -0.5) as differentially expressed. We exercised caution in selecting gene expression datasets to ensure they would not compromise the sensitivity of the ML model. So, the GSE37772 and GSE111175 datasets are excluded from performing machine learning analysis. GSE42133 is examined, identified as relevant, and selected to build the XAI model.

### Shapley additive explanations

**Fig. 6:**
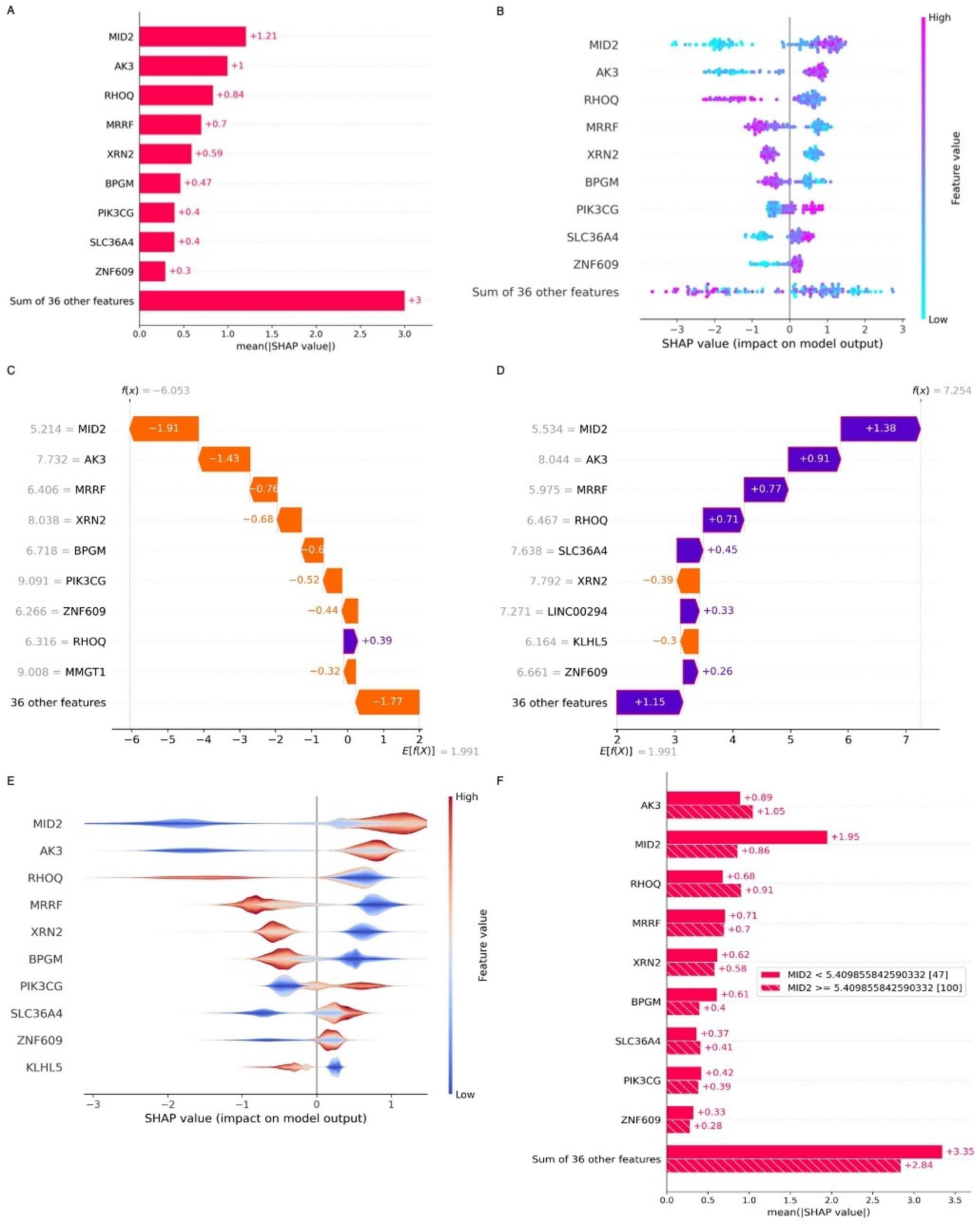
(**A**) Global bar plot with mean SHAP value of each gene denoting its contribution to the predictions. (**B**) Beeswarm plot with SHAP value (negative value represents the genes influencing the prediction towards “Control,” whereas the positive value indicates the genes predicting the sample as “ASD” under different expression levels of each gene). (**C, D**). A local bar plot was generated for a random control sample (left) and an ASD sample (right) taken from the dataset to interpret the prediction. The results show that the increased expression levels of MID2 (5.534) by the model are predicted as ASD, whereas the expression levels are decreased on the samples predicted as control (5.214). (**E**) Summary plot of top 10 genes influencing the predictions. (**F**) Cohort plot revealing the most influential gene predicting the samples with the highest accuracy (MID2). The values MID2 < 5.40 indicate the lower expression levels of the gene influencing the predictions of samples as control (47 out of 56) and MID2 > 5.40 as ASD (100 out of 91) with nine misclassifications

The initial DEGs of GSE42133 containing 172 gene biomarkers that satisfied the corresponding criteria logFC > 0.5 or logFC < -0.5 and adj. p-value < 0.01 were scrutinized with a recursive feature elimination algorithm (RFE), a wrapper-based feature selection method. It selects the most relevant features in a dataset by iteratively eliminating less important features based on a specified model’s performance. The subset generated by the algorithm is trained with support vector machines to determine the scores of each set. The resultant subset with the best performance contains 46 genes as candidate markers of ASD. This 46-feature subset is further trained in the next process using an extreme gradient boosting algorithm (XGBoost) for XAI model preparation. The trained model is fed as an input into the SHAP framework to perform the interpretation, thereby understanding the decision of every prediction made by the black-box model. The overall importance of each feature based on its contribution towards the prediction is depicted in Fig. 6A-B, containing a global bar and bee-swarm plot. The higher mean SHAP value (mean absolute value) denotes that such features significantly impact the predictions. *MID2* stands on top with the highest score of + 1.21, followed by *AK3* (+ 1) and *RHOQ* (+ 0.84) in three consecutive scores. The bee-swarm plot provides insight into the genes with positive and negative SHAP values. The positive SHAP value represents the influence of genes irrespective of the feature value (increased/decreased expression levels) to “ASD,” and the negative SHAP value denotes the “control” prediction. The data points visualized in the beeswarm plot in “cyan” show decreased expression levels, and the “violet” indicates increased expression levels for the particular gene for all the samples. In Fig. 6C-D, the parameter E[f(X)] is the baseline, and f(x) is the value predicted by our model. The values on the x-axis assigned to each gene are the actual expression values on the particular sample. In comparing the results between the two samples of ASD and control, the genes *MID2* and *AK3* clearly explain that the increased expression levels are influencing the predictions of ASD and the low expression levels to control. Figure 6E-F represents the summary and cohort plots generated based on the Shapley values. The cohort plot clearly shows that the increased expression levels of *MID2* predict the samples as ASD, whereas the lower levels are classified into normal samples. The summary plot is the smooth version of the bee-swarm plot with a violin-like representation.

## Discussion

Rapid advancements in computing have made their way into high throughput bioinformatics strategies, with AI and advanced ML models leading the charge in bioinformatics and computational biology. Enhanced data processing predictive modeling is now employed to develop accurate and precise therapeutic strategies (Subramanian et al. 2020; Bonkhoff and Grefkes 2022). This work identified DEGs across multiple GEO datasets related to ASD. Subsequent bioinformatics analyses were performed to investigate these DEGs’ putative functions and molecular interactions. Furthermore, XAI was employed to identify candidate biomarker genes for ASD. Cell type-specific gene expression profiling analyses are useful approaches to identifying genes specifically expressed in certain cell types and play an important role in ASD (Raudvere et al. 2019). However, gene expression in peripheral blood cells is very sensitive to stress so gene expression patterns may be altered during cell isolation and purification (Pascual et al. 2010). This study used five different datasets from human blood studies (Table 2). Figure 2 A illustrates comparable expression levels between control (healthy) and autistic individuals across the analyzed datasets.

**Table 2:** The Top functional enrichment categories

Category	Term	Count	%	P-value	Benjamini	FDR
UP_TISSUE	Brain	165	48.25	0.03	0.96	0.96
CHROMOSOME	17	30	8.77	0.01	0.19	0.19
CHROMOSOME	19	31	9.06	0.02	0.28	0.28
UP_KEYWORDS	Alternative splicing	208	60.82	0.00	0.02	0.02
UP_KEYWORDS	Phosphoprotein	175	51.17	0.00	0.00	0.00
UP_KEYWORDS	Cytoplasm	116	33.92	0.00	0.00	0.00
UP_KEYWORDS	Acetylation	84	24.56	0.00	0.02	0.01
GAD_DISEASE_CLASS	CANCER	82	23.98	0.00	0.03	0.03
GAD_DISEASE_CLASS	PHARMACOGENOMIC	68	19.88	0.01	0.06	0.05
GAD_DISEASE_CLASS	NEUROLOGICAL	67	19.59	0.05	0.13	0.12
CYTOBAND	Xq13.1	4	1.17	0.02	1.00	1.00
CYTOBAND	22q13.1	4	1.17	0.05	1.00	1.00

Furthermore, we identified shared genes and loci, pathogenic SNPs, distributions of SNP frequencies, and the chromosomal locations of these SNPs. We also mapped the PPI network for these associated genes (Fig. 3). Through comprehensive analysis, we pinpointed the genes most closely related to ASD and related conditions. Subsequent enrichment analysis was conducted to understand the functional implications of these identified genes. Our analysis identified 19 upregulated and 10 downregulated genes (Fig. 2B). The following genes exhibited up-regulation: *UTY* (ubiquitously transcribed tetratricopeptide repeat-containing, Y-linked), *GUCY1A3* (Guanylate cyclase soluble subunit alpha-3), *NR2F2* (nuclear receptor subfamily 2 group F member 2), and *GUCY1B3* (Guanylate cyclase soluble subunit beta-1).

Conversely, the genes *CYP20A1* (cytochrome P450 family 20 subfamily A member 1) and *CTBP1* (C-terminal binding protein 1) were significantly down-regulated. A comparison with text mining results from extant literature on ASD indicates that these genes were not previously associated with ASD. As a result, they may represent novel candidate genes potentially implicated in ASD pathogenesis.

Upon further annotation of the 342 DEGs with an adjusted p-value of 0.001, chromosomes 17 and 19 were identified to harbor the highest number of these DEGs (Table 1). Numerous cytogenetic investigations have revealed anomalies on chromosomes 17 and 19, including duplications, deletions, and inversions, within regions housing potential ASD-associated genes (Miles 2011; Butler et al. 2015). Moreover, 22q13.1 and Xq13.1 were found previously as chromosome locations for many related ASD genes (Butler et al. 2015). The q arm at position 13.1 of chromosome 22 had the highest number of ASD-associated genes compared to other locations. Also, it has been discovered that new microduplication in Xq13.1 is linked to autism and speech delay (Gumus 2019). In the current study, genes associated with oncological, pharmacogenomic, and neurological domains exhibited the highest frequency of investigation (Table 1) (Crawley et al. 2016; Xiong et al. 2019). Genes associated with cancer processes were found belong to various biological functions, including cellular proliferation (e.g., C-terminal binding protein 1 - *CTBP1*), cell adhesion (e.g., integrin subunit alpha 2 - *ITGA2* and cadherin 1 - *CDH1*), growth and development (e.g., platelet-derived growth factor subunit A - *PDGFA*), and cell death promotion (e.g., axin 1 - *AXIN1*). Pharmacogenomics studies have systematically investigated the associations between genetic variants, therapeutic responses, and adverse reactions. Historically, the primary focus has been the study of antidepressants, antipsychotics, and stimulants, the predominant pharmacological classes utilized in treating ASD (Brown et al. 2017).

Our study revealed that genetic polymorphisms and alternative splicing significantly influence our datasets related to ASD. Unlike conditions such as Fragile X Syndrome, defined by specific gene mutations, ASD does not have unique polymorphisms that can act as definitive biomarkers for prediction. This complexity results in genetic variants across numerous genes associated with ASD risk. It is also worth noting that while ASD prevalence is rising, the exact rates, especially within families already affected by ASD, require further verification (Steinman 2018). In addition, evidence suggests that disruption of the normal splicing sites can lead to many human diseases, like ASD (Cieply and Carstens 2015; Quesnel-Vallières et al. 2016).

In our work, the caffeine metabolism pathway featured the most significant proportion of involved genes (Fig. 4A). Research into metabolic profiles in children with ASD has underscored the significance of caffeine metabolism as a central pathway when comparing typically healthy children with those who have ASD (Rangel-Huerta et al. 2019). For our GO analysis, we focused on the biological processes (BP) that encompass stimulus, signaling, and development or regulation of the nervous-related processes (Fig. 4B). As a result of our investigation, it became evident that most of the genes examined in our study play a significant role in biological processes related to Autism Spectrum Disorder (ASD). We sought to pinpoint common genes between two distinct groups: 19 genes that exhibited increased activity and 53 genes associated with the development or regulation of the nervous system, as revealed by our biological analysis.

We identified seven shared genes between these groups: *HOXB3, NR2F2, MAPK8IP3, PIGT, SEMA4D, and SSH1*. Strikingly, six of these genes, excluding *PLK2*, were absent from the results obtained through text mining. Therefore, these six genes represent promising candidates not previously associated with ASD and could play pivotal roles in the disorder.

We identified 1,286 pathogenic SNPs. Most of these SNPs were distributed among disorders with pathways that are either directly linked or related to ASD. Specifically, they were found in cancer (58.55%), mental or intellectual disorders (16.17%), neuronal diseases (7.78%), Noonan syndrome (6.45%), developmental delays or autism (5.21%), brain-related conditions (3.65%), and mitochondrial disorders (2.18%) (Fig. 4C). The significant representation of genes in cancer pathways corroborates a previous study that highlighted shared pathways, risk genes, and drug targets between cancer and ASD (Crawley et al. 2016). While many diseases can be categorized as mental or neuronal, there is a recognized overlap among them (Sullivan et al. 2019). This underscores the significance of our ASD-related findings and suggests that the identified SNPs should be considered strongly related to the pathogenesis of ASD.

Furthermore, 14 pathogenic SNPs were directly associated with ASD. We noticed these pathogenic SNPs were the most found in chromosome 10 and chromosome X, with 7 and 5 pathogenic SNPs, respectively. Many studies linked many genes on chromosomes X and 10 with ASD. On the other hand, we found that chromosome X has the highest number of pathogenic SNPs in our study that are related to delay or autism and intellectual or mental diseases (Fig. 5A). A recent study on analysis of the genetics related to ASD and intellectual disability found many genes on the X chromosome (Chiurazzi et al. 2020). Moreover, as it is known, the ASD ratio is higher in males than females. Therefore, many theories explain the relationships between the genes on chromosome X and the high ratio in males (Baron-Cohen et al. 2011). In addition, for chromosome 10, a study proved that some genes affect the abilities of autistic (Chapman et al. 2011).

The biomarker chart displays potential biomarkers that exhibit responsiveness to targeted therapies designed for ASD (Fig. 5B). As seen in Fig. 5B, the presence of the highest number of pathogenic SNPs in FGFR inhibitor suggests that it may serve as a promising potential biomarker for responsiveness to targeted therapies for ASD in further studies. Previous studies found that interruption of signaling of FGFR pathways could act as a possible function in ASD’s molecular pathology (Wu et al. 2016, 2020). As can be seen in Fig. 5C, the missense variants are the most frequent in ASD-diseased patients, and this is in agreement with a recent paper that sequenced a large number of autistic individuals (6430) and found the highest frequency was for missense in exons of protein-coding regions (Satterstrom et al. 2020). Another recent study on missense variants in ASD found that many missense variants in autistic individuals damage central proteins and interactions (Chen et al. 2020).

While conducting experiments using the five datasets to identify biomarkers of DEGs for the preparation of the XAI pipeline, we identified two specific genes, *RPS4Y1* and *KDM5D*, exhibiting statistical significance for the GSE37772 according to the dataset selection criteria. The dataset GSE111175 does not show any DEGs. The *RPS4Y1 and KDM5D* are Y-linked chromosome genes. The *RPS4Y1* regulates trophoblast cell migration and invasion through the STAT3epithelial–mesenchymal transition pathway (Chen et al. 2018), and emerging research hints at its potentially pivotal role in neurogenesis (Khani et al. 2022). The *KDM5D* contains coding information for a protein featuring zinc finger domains, and a small peptide derived from this protein serves as a minor histocompatibility antigen. Hatch et al. (2021) and Zamurrad et al. (2018) highlighted the significance of members within the *KDM5* gene family in neurodevelopment. Several studies have explored the association of the KDM5 gene family with ASD and identified pathological significance (El Hayek et al. 2020). The *RPS4Y1*, the chromosome Y encoded gene and also an inhibitor of STAT3 signaling, is identified as a contributor of ASD specific to male predominance. The GSE42133 containing 172 gene biomarkers, further scrutinized into 46 with RFE, is trained with the XAI model to reveal the key marker discriminating the ASD and control samples.

The XAI model identified *MID2* as a key biomarker differentiating control from ASD groups. Elevated *MID2* gene expression levels have been associated with a potential predictive factor for ASD. The MID2 protein, known as Midline-1-interacting protein 2, is encoded by the *MID2* gene located on the X chromosome in humans (Geetha et al. 2014). Research has unveiled the biological significance of MID2, which is associated with conditions such as intellectual disability. With a SHAP value of + 1.21, this biomarker is linked to diseases like autism affecting cognitive and motor functions. MID2 is a member of the E3 ubiquitin ligases protein family, which controls cellular activities and aids in protein breakdown. The ubiquitin-proteasome pathway, which is responsible for the selective degradation of proteins within cells, specifically involves the MID2 (Bonini et al. 2017). Mutations or abnormalities in the *MID2* gene have been associated with various genetic disorders. One well-known disorder associated with *MID2* is Opitz G/BBB syndrome type 1 (FG syndrome), characterized by developmental and intellectual disabilities (Ferrentino et al. 2007). Children initially diagnosed with ASD often display characteristics of FG syndrome. Exploring the protein and its associated biological pathways will provide fresh insights into the connection between the gene and ASD (Lasser et al. 2018).

## Conclusion

In this study, we used comprehensive bioinformatics, advanced machine learning techniques, and XAI methodologies to unravel the complex genetic landscape of ASD. The rigorous analysis of multiple GEO datasets, alongside in-depth bioinformatics assessments, led to the identification of a significant number of DEGs that are associated with ASD. We compared our findings with similar studies to identify common trends and further elucidate certain aspects, specifically the pathogenesis and risk factors associated with SNPs. Our XAI model identified *MID2* as a potential clinical biomarker for ASD. It is important to note that our analysis had limitations stemming from the unavailability of detailed clinical data, which limited the potential genotype-phenotype correlation. In the future, the analysis of multimodal genetic datasets of many patients integrated with clinical information promises to unlock profound insights into the molecular and clinical pathogenesis of ASD. This will provide a comprehensive understanding of gene functionality, gene loci, observed SNPs, dysregulated pathways in ASD, and their impact on clinical measures. Ultimately, these insights will facilitate the development of more accurate treatment approaches for ASD.
